# Risk factors analysis and prediction model construction of hospital-acquired pneumonia after traumatic brain injury

**DOI:** 10.3389/fneur.2025.1518599

**Published:** 2025-07-10

**Authors:** Xiao-Cong Wei, Yi-Zi Zhang, Min Guo, Hai-Bo Tong, Yong-Hong Wang, Xiao-Qin Wang, Hong-Ming Ji, Bin Ren, Hao Wu

**Affiliations:** ^1^Shanxi Bethune Hospital, Shanxi Academy of Medical Sciences, Tongji Shanxi Hospital, Third Hospital of Shanxi Medical University, Taiyuan, China; ^2^Shanxi Provincial People's Hospital, Taiyuan, China

**Keywords:** traumatic brain injury, pneumonia, prediction model, risk factors analysis, hospital-acquired pneumonia (HAP)

## Abstract

**Background:**

Hospital-acquired pneumonia (HBP) is a common and serious infections disease that affects the patients with traumatic brain injury (TBI). Severe pneumonia can lead to high mortality and morbidity in TBI patients. Therefore, it is important to investigate the risk factors and develop a prediction model for HBP following TBI.

**Methods:**

The clinical data of 285 patients with TBI, admitted to Shanxi Bethune Hospital and Shanxi Provincial People’s Hospital, were collected. Patients were divided into two groups based on the presence or absence of pneumonia. Risk factors for HBP were identified, a predictive model was constructed, and its performance was validated.

**Results:**

Significant differences were observed between the pneumonia and non-pneumonia groups regarding several factors, including age, history of diabetes, smoking history, white blood cell count, platelet count, albumin levels, Glasgow Coma Scale (GCS) score upon admission, thoracic trauma, craniocerebral surgery, and the need for tracheal intubation post-admission (*p* < 0.05). Among these, age, smoking history, thoracic trauma, white blood cell count, albumin levels, and admission GCS score were identified as independent risk factors for HBP following TBI. The predictive model based on these six factors demonstrated high accuracy.

**Conclusion:**

Age, smoking history, thoracic trauma, white blood cell count, albumin levels, and admission GCS score are independent risk factors for HBP after TBI. The predictive model developed based on these factors shows strong predictive accuracy and clinical utility.

## Introduction

Traumatic brain injury (TBI) refers to brain damage caused by trauma, car accidents, sports injuries, etc., and is a major global health problem (1). Over the past decade, the number of deaths resulting from TBI continues to rise significantly (2–4). Severe TBIs, such as brain contusions and diffuse axonal injuries, greatly impair brain function, often leading to late-onset bleeding, altered consciousness, and physical disabilities. These conditions are frequently accompanied by complications such as dysphagia, vomiting, impaired cough reflex, and an inability to clear oral secretions, all of which significantly increase the risk of aspiration and subsequent pneumonia (5). Recent studies have demonstrated that patients with TBI exhibit increased blood glucose variability, likely due to the traumatic stress response. This elevated blood glucose level is closely associated with a higher incidence of lung infections, contributing to increase in-hospital mortality and being positively correlated with poor clinical outcomes (6). In addition, studies have shown that the incidence of pneumonia in TBI patients can exceed 60%, making it an independent risk factor for poor prognosis (7). Severe pneumonia has even become one of the leading causes of death in TBI patients, not only affecting patient outcomes but also imposing a substantial financial burden on families (8, 9). Effective management of TBI requires not only treating the injury itself but also addressing complications, particularly pneumonia. Early identification of patients at high risk for pneumonia is therefore crucial in the care of TBI patients.

To comprehensively evaluate the risk factors for pneumonia following TBI and develop an early prediction model, we collected clinical data from 285 TBI patients treated at Shanxi Bethune Hospital and Shanxi Provincial People’s Hospital between January 2020 and May 2022. The aim of this study was to improve secondary prevention and management of hospital-acquired pneumonia (HBP) in patients with TBI.

## Methods

### Study design and population

A retrospective study was conducted on TBI patients admitted to Shanxi Bethune Hospital and Shanxi Provincial People’s Hospital between January 2020 and May 2022. The inclusion criteria were: (1) a confirmed history of trauma at admission; (2) brain imaging on admission showing traumatic changes such as brain contusion, laceration, skull fracture, or intracranial hematoma; (3) patients aged over 18 years. The exclusion criteria were: (1) unstable vital signs upon admission; (2) presence of serious underlying diseases that preclude the patient from tolerating trauma or surgery; (3) TBI caused by other primary neurological conditions, such as hypertensive cerebral hemorrhage; (4) pneumonia diagnosed at admission (including aspiration pneumonia, bacterial pneumonia, viral pneumonia, COVID-19, etc.); (5) refusal by the patient or their family to cooperate with treatment; (6) presence of other factors that may affect the study’s results.

A total of 285 TBI patients were included in this study, following the approval of more than three years of experience by neurosurgeons and the review by one neurosurgical director. The study protocol was approved by the Ethics Committee of Shanxi Bethune Hospital (YXLL-2022-124).

### Data collection

Data collection for all enrolled patients was conducted by neurosurgery specialists and specialist nurse. General information gathered included gender, age, height, weight, smoking history, drinking history, and medical history. Clinical data comprised the Glasgow Coma Scale (GCS) score at admission, presence of chest trauma, tracheal intubation status, imaging examination results, routine blood tests, coagulation function, liver and kidney function, blood electrolytes, blood glucose levels, and serum albumin levels. All laboratory test results were obtained within 24 h of patient admission.

### Diagnosis and case grouping of pneumonia

The diagnosis of pneumonia was made according to the Chinese guidelines for the diagnosis and treatment of HBP in adults and ventilator-associated pneumonia (2018 edition) (10). Pneumonia was diagnosed based on chest X-ray or CT findings showing new or progressive infiltrative shadows, consolidation, or ground-glass opacities, combined with two or more of the following three clinical symptoms: (1) fever with a temperature > 38°C; (2) purulent airway secretions; (3) peripheral blood leukocyte count > 10 × 10^9^/L or < 4 × 10^9^/L. Additionally, patients suspected of having ventilator-associated pneumonia (except for those who required ventilator support during surgery and anesthesia) and those diagnosed with pneumonia at admission (such as COVID-19 or other viral pneumonia) were excluded from the study.

It is important to note that this study focuses on early-onset pneumonia in TBI patients, and all risk factors were collected within the first 24 h of patient admission. Therefore, only patients diagnosed with pneumonia within 5 days of admission were included in the study. Pneumonia occurring after 5 days was considered to have missed the opportunity for early intervention, and these patients were excluded from the analysis. Pneumonia diagnoses were confirmed by two neurosurgery specialists following these criteria. In cases where diagnostic difficulties or disagreements arose, consultations were conducted with respiratory or critical care physicians with senior titles to resolve the issues and reach a final diagnosis. Ultimately, of the 285 TBI patients included in the study, 138 were diagnosed with pneumonia, while 147 were confirmed to be pneumonia-free.

### Predictor variables

Refer to Table 1 for details on the indicators observed in the study. All measurements were taken on the first day after admission. Chest trauma primarily included rib fractures, hemopneumothorax, pulmonary contusion, and laceration. Chronic lung diseases encompassed conditions such as chronic bronchitis, interstitial pneumonia, and chronic obstructive pulmonary disease (COPD). Tracheal intubation and craniocerebral surgeries performed within the first 24 h of admission were also recorded.

**Table 1:** General and clinical data of TBI patients.

Variable	Total (*n* = 285)	Pneumonia		Statistic	*P* value
		None (*n* = 138)	Yes (*n* = 147)		
Age, years	51.70 ± 16.31	48.42 +/− 18.15	54.78 ± 13.72	T = 3.321	0.001
Hemoglobin, g/dL	136.00 (121.00–146.00)	137.00 (124.00–147.00)	135.00 (113.00–144.00)	Z = 1.943	0.052
White blood cell count, 10^9^/L	12.40 (8.90–16.10)	10.80 (7.70–14.57)	14.00 (10.15–17.00)	Z = 4.217	The < 0.001
The platelet count, 10^9^/L	201.00 (167.00–244.00)	212.50 (181.25–248.00)	196.00 (141.50–236.00)	Z = 2.921	0.003
Serum creatinine, mg/dL	72.20 (65.30–82.10)	71.85 (65.55 81.75)	72.80 (63.90–82.35)	Z = 0.205	0.838
Urea nitrogen, mg/dL	5.10 (4.10–6.00)	4.95 (3.90–6.00)	5.30 (4.30–6.05)	Z = 1.199	0.231
Activated partial prothrombin time, s	29.60 (27.00–31.50)	29.85 (27.63–31.37)	29.00 (26.75–31.80)	Z = 1.379	0.168
Prothrombin time, s	11.60 (11.00–12.20)	11.50 (10.90–12.07)	11.70 (11.05–12.35)	Z = 1.598	0.11
Alanine aminotransferase, IU/L	22.60 (15.30 33.10)	20.20 (15.27–31.68)	24.20 (15.50–33.55)	Z = 1.114	0.265
Aspertate aminotransferase, IU/L	24.00 (17.60–33.40)	23.35 (17.52–31.73)	24.60 (17.75–34.70)	Z = 0.715	0.475
Blood albumin, g/L	39.50 (35.80–42.20)	40.50 (37.50 42.68)	38.50 (33.60–41.80)	Z = 3.552	The < 0.001
Serum sodium, mmol/L	137.60 (135.60139.50)	137.80 (135.22139.57)	137.50 (135.80–139.50)	Z = 0.063	0.95
Potassium ion, the tendency for L	3.71 (3.40–4.00)	3.73 (3.40 4.00)	3.70 (3.38–4.01)	Z = 0.009	0.993
Blood sugar, the tendency for L	7.10 (6.20 8.93)	7.10 (6.10–9.06)	7.00 (6.31–8.90)	Z = 0.055	0.956
GCS at admission	11.00 (7.00–14.00)	13.00 (10.00–14.00)	8.00 (5.50–12.00)	Z = 6.228	The <0.001
GCS at discharge	12.00 (8.00–15.00)	14.00 (12.00–15.00)	8.00 (6.00–13.00)	Z = 7.454	<0.001
Length of ICU stay (days)	7 0.00(5.00–9.00)	7.00(6.00–9.00)	6.00(5.00–8.00)	Z = 2.382	0.017
Length of hospital stay (days)	18 0.00(12.00–29.00)	15.00(12.00–17.00)	19.00(12.00–31.00)	Z = 2.183	0.029
Gender, *n* (%)				Squared = 0.992	0.319
female	65 (22.81)	35 (25.36)	30 (20.41)		
male	220 (77.19)	103 (74.64)	117 (79.59)		
BMI>23 9, *n* (%)				Squared = 1.655	0.198
No	209 (73.33)	106 (76.81)	103 (70.07)		
Yes	76 (26.67)	32 (23.19)	44 (29.93)		
History of alcohol consumption, *n* (%)				Squared = 0.046	0.83
No	209 (73.33)	102 (73.91)	107 (72.79)		
Yes	76 (26.67)	36 (26.09)	40 (27.21)		
Smoking history, *n* (%)				Squared = 8.154	0.004
No	174 (61.05)	96 (69.57)	78 (53.06)		
Yes	111 (38.95)	42 (30.43)	69 (46.94)		
History of hypertension, *n* (%)				Squared = 1.833	0.176
No	202 (70.88)	103 (74.64)	99 (67.35)		
Yes	83 (29.12)	35 (25.36)	48 (32.65)		
History of diabetes mellitus, *n* (%)				Squared = 7.448	0.006
No	251 (88.07)	129 (93.48)	122 (82.99)		
Yes	34 (11.93)	9 (6.52)	25 (17.01)		
Chest trauma, *n* (%)				Squared = 12.624	The <0.001
No	232 (81.4)	124 (89.86)	108 (73.47)		
Yes	53 (18.6)	14 (10.14)	39 (26.53)		
Chronic lung disease, *n* (%)				Squared = 0.143	0.706
No	214 (75.09)	105 (76.09)	109 (74.15)		
Yes	71 (24.91)	33 (23.91)	38 (25.85)		
Craniocerebral surgery, *n* (%)				Squared = 6.191	0.013
No	178 (62.68)	96 (70.07)	82 (55.78)		
Yes	106 (37.32)	41 (29.93)	65 (44.22)		
Endotracheal intubation, *n* (%)				Squared = 32.000	The <0.001
No	164 (57.54)	103 (74.64)	61 (41.50)		
Yes	121 (42.46)	35 (25.36)	86 (58.50)		

### Statistical analysis

Data processing and analysis were performed using SPSS 26.0 statistical software. Categorical data were expressed as relative frequencies (%) or rates (%), and the chi-square test was used for comparisons. Normally distributed continuous data were expressed as mean ± standard deviation (x ± s), with group comparisons performed using the t-test. Non-normally distributed data were presented as median and interquartile range [M (P25, P75)], and group differences were analyzed using the Mann–Whitney U test.

### Logistic regression and prediction model construction

Logistic regression was conducted to analyze the risk factors for pneumonia in TBI patients, with pneumonia status (presence or absence) as the dependent variable and the statistically significant factors as independent variables. The *p* value < 0.05 was considered statistically significant. Multicollinearity between variables was assessed using the variance inflation factor (VIF), with VIF values > 10 indicating the presence of multicollinearity. To assess the predictive value of independent and combined risk factors for pneumonia, receiver operating characteristic (ROC) curves were drawn. A nomogram was subsequently constructed using R software to visually represent the prediction model.

## Results

### Characteristics of the study population

Significant differences were observed between the two groups in terms of age, white blood cell count, platelet count, serum albumin levels, GCS score, smoking history, diabetes history, chest trauma, craniocerebral surgery, and endotracheal intubation. No significant differences were found for the remaining variables. Additionally, we observed that the median GCS of TBI patients who did not develop pneumonia increased from 13 at admission to 14 at discharge, whereas the median GCS of patients who developed pneumonia remained at 8 both at admission and discharge. Furthermore, TBI patients who did not develop pneumonia had shorter ICU stays and overall hospital stays compared to those who developed pneumonia (*p* < 0.05). These findings demonstrate the adverse impact of pneumonia on the prognosis of TBI patients. Detailed baseline data are provided in Table 1.

### Risk factors analysis results

Multivariable logistic regression analysis was conducted to explore the risk factors for pneumonia, with pneumonia as the dependent variable and the factors showing statistically significant differences as independent variables. Due to the large number of independent variables, a two-way stepwise regression approach was employed. Ultimately, six factors of age, smoking history, chest trauma, white blood cell count, serum albumin levels, and GCS score were identified as independent risk factors for the development of pneumonia in TBI patients (Table 2). There is no multicollinearity between the variables (VIF < 10).

**Table 2:** Logistic regression analysis of risk factors for pneumonia in TBI patients.

Variable	B	Standard errors	Wald	*P* value	OR	95% CI	VIF
Age	0.023	0.009	5.967	0.015	1.023	1.004–1.042	1.311
Smoking history	0.59	0.284	4.332	0.037	1.804	1.035–3.145	1.179
Chest trauma	1.085	0.372	8.49	0.004	2.960	1.426–6.141	1.099
White blood cell count	0.08	0.029	7.533	0.006	1.084	1.023–1.147	1.431
Blood albumin	0.082	0.027	9.035	0.003	0.921	0.873–0.972	1.235
GCS score	0.151	0.039	14.633	< 0.001	0.860	0.796–0.929	1.275

### Prediction model construction

**Figure 1:**
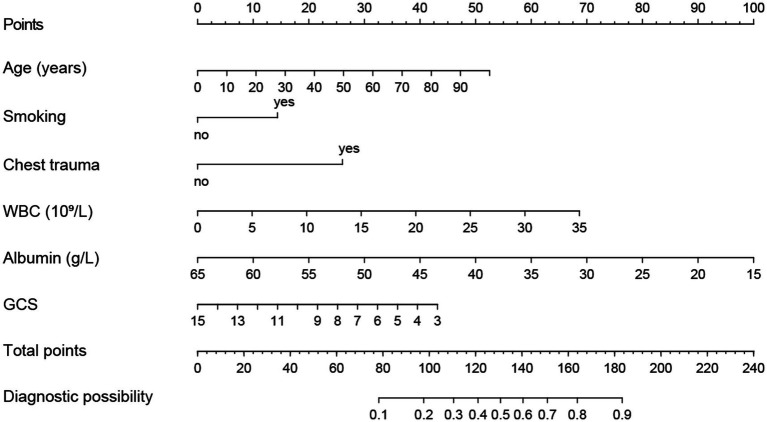
Nomogram for predicting the incidence of pneumonia associated with TBI.

**Figure 2:**
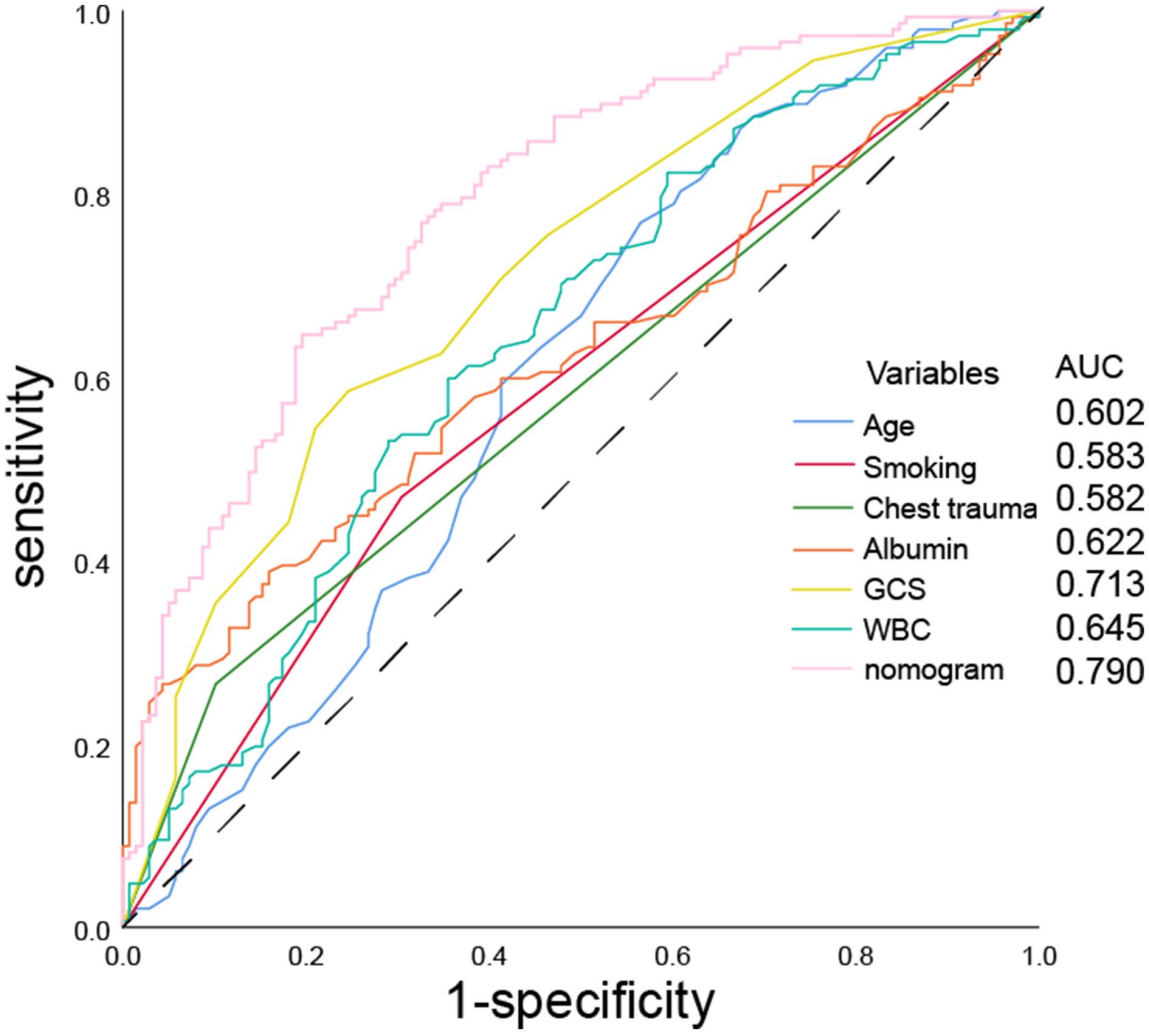
ROC curves of age, smoking history, chest trauma, white blood cell count, serum albumin, GCS score, and nomogram for predicting the incidence of pneumonia.

**Figure 3:**
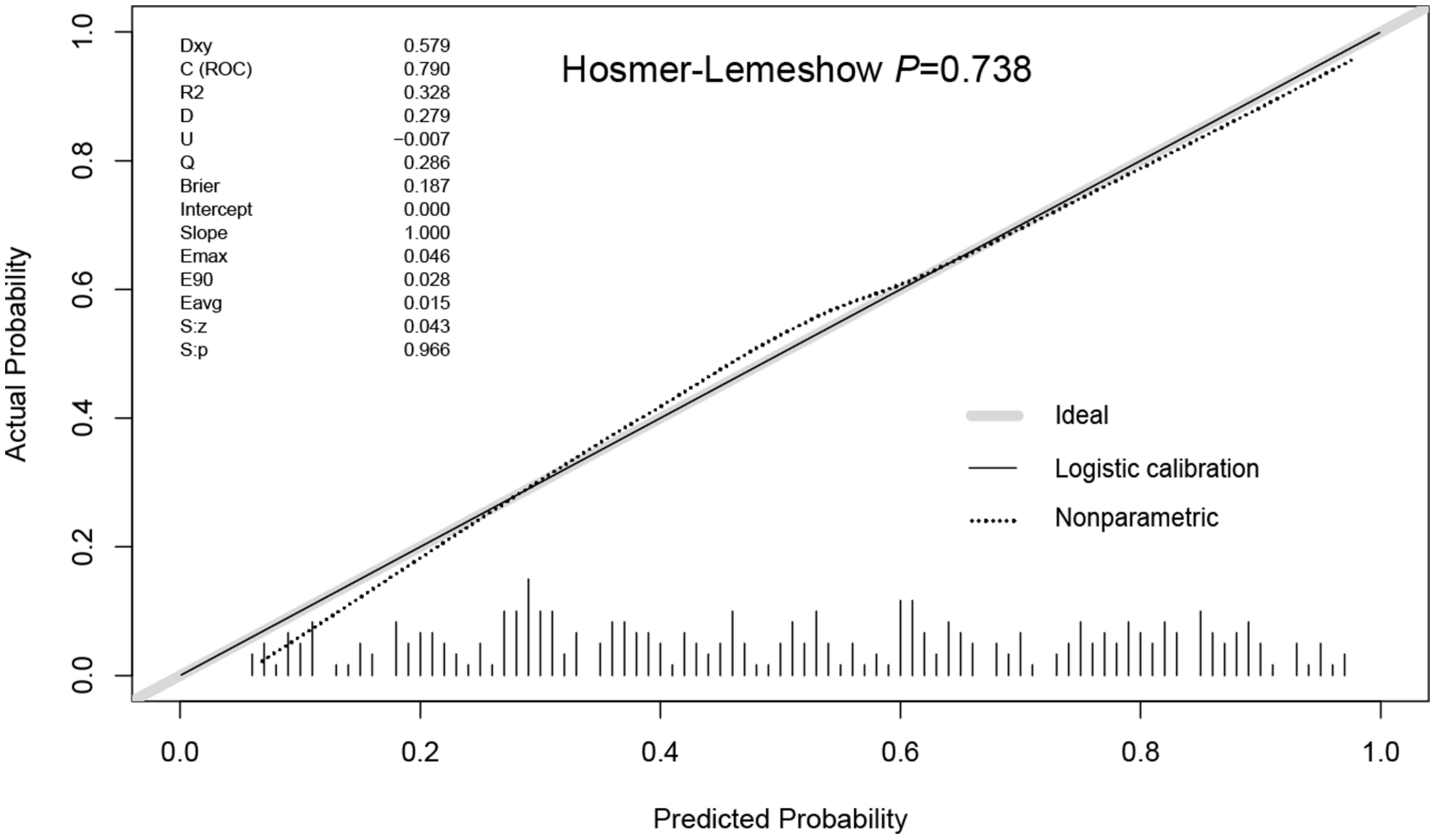
Calibration plot of the prediction model for TBI associated pneumonia risk nomogram.

**Figure 4:**
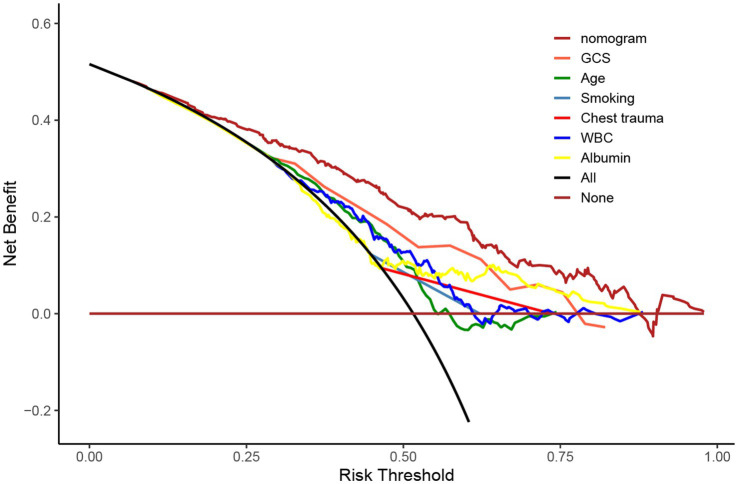
DCA curves of age, smoking history, chest trauma, white blood cell count, serum albumin, GCS score, and nomogram for predicting incidence of pneumonia.

Using R software, we analyzed factors including age, smoking history, chest trauma, white blood cell count, blood albumin levels, and GCS score to develop a nomogram as a predictive model for pneumonia associated with TBI, as illustrated in Figure 1. The performance of the nomogram was tested using ROC curve, calibration curve, and DCA. The AUC of the nomogram was 0.790, which is higher than that of individual factors (AUC = 0.602, 0.583, 0.582, 0.622, 0.713, 0.645, 0.790; Figure 2), indicating a high discriminatory ability. The calibration curve closely followed the standard curve, and the *p*-value from the Hosmer-Lemeshow test was 0.738 (Figure 3), which indicates good calibration of the nomogram. The DCA showed that using the nomogram resulted in better clinical decision-making efficiency compared to using individual factors. Furthermore, the nomogram demonstrated good clinical decision-making efficiency across different risk thresholds (Figure 4).

## Discussion

According to the U.S. National Institute on Disability and Rehabilitation, despite being the accepted standard for rehabilitating patients with moderately TBI, the mortality rate remains 2.2 times higher than that of the general population, with a reduced life expectancy of 6.6 years. Furthermore, TBI patients are 44 times more likely to die from pneumonia compared to the general population (11). Pneumonia is identified as an independent predictor of poor prognosis in severe TBI patients five years post-discharge (8). Severe pneumonia, particularly acute respiratory distress syndrome (ARDS), can lead to mortality in TBI patients (12). The risk of pneumonia in TBI patients is heightened due to neurological impairments, which can include altered consciousness, difficulty swallowing, compromised vomiting reflex, and impaired cough reflex, as well as ventilator-associated pneumonia.

Recent studies have revealed a complex interplay between the immune and nervous systems regarding TBI and pneumonia, suggesting a significant relationship between these two conditions (3, 13, 14). Following TBI, abnormalities such as dysregulated adrenal hormone secretion, activation of damage-associated molecular patterns (DAMPs), and cytokine release contribute to acute lung injury, pulmonary edema, and even ARDS, thereby exacerbating the peripheral inflammatory response. Additionally, inflammatory mediators may breach the damaged blood–brain barrier and the brain’s lymphatic-like system, influencing immune cells within brain tissue and further increasing blood–brain barrier permeability. This cascade results in an aggravated central inflammatory response, leading to brain edema (3). Simultaneously, TBI can diminish the functionality of immune cells, such as neutrophils and natural killer (NK) cells, through various mechanisms. This results in decreased production of pro-inflammatory cytokines like interleukin (IL)-1β and tumor necrosis factor (TNF)-*α*, while increasing the production of the anti-inflammatory cytokine IL-10, ultimately leading to immunosuppression and the exacerbation of pneumonia (13, 14).

The treatment of pneumonia typically involves the use of sensitive antibiotics, along with encouraging patients to cough and mobilize to facilitate inflammation absorption and recovery. However, in the context of TBI, neurological damage from trauma often leads to prolonged disturbances in consciousness, impairing the recovery of autonomic functions. This significantly extends the recovery period for pneumonia and increases the risk of antibiotic resistance among pathogenic bacteria, complicating treatment efforts. Therefore, emphasis should be placed on preventing pneumonia secondary to TBI. Early initiation of anti-infection treatments and high-quality nursing care for at-risk pneumonia groups can effectively reduce pneumonia-related mortality. Commonly used serum inflammatory markers for predicting HBP include procalcitonin (PCT), C-reactive protein (CRP), and interleukin-6 (IL-6). However, relying on a single blood marker may be overly simplistic. Consequently, in our study, we included a comprehensive array of indicators for predicting pneumonia occurrence, encompassing general demographic data, medical history, physical examination findings, imaging results, and blood inflammatory markers. This approach enabled us to construct a robust predictive model. The pneumonia prediction model, which incorporates factors such as age, smoking history, chest trauma, white blood cell count, serum albumin levels, and GCS, demonstrates high predictive accuracy. Each of these factors provides a comprehensive assessment of the actual lung injury in TBI patients and offers valuable insights into the severity of pneumonia.

Compared to previous studies (7), the factors included in our analysis were expanded to include serum albumin, which is consistent with clinical observations. In clinical practice, particularly in ICU settings, albumin is commonly used to assess a patient’s baseline nutritional status. Hypoalbuminemia is frequently observed in populations such as the elderly and malnourished individuals, who have a reduced ability to withstand traumatic insults and are therefore more susceptible to infections. Additionally, some factors that are considered high-risk for pneumonia in other studies, such as chronic pulmonary disease, were not included in our analysis. This may be attributed to the relatively small sample size and potential bias in the collection of patient histories, which is also a limitation of this study as a retrospective analysis. Furthermore, the lack of external validation data is another limitation of this study.

In conclusion, pneumonia is one of the most prevalent and serious complications in patients with TBI. Early diagnosis and targeted prevention and treatment strategies for high-risk groups are essential. The establishment of a pneumonia disease prediction model offers a reliable method for assessing the risk of HBP in TBI patients, facilitating effective secondary prevention and treatment measures.

## Data Availability

The original contributions presented in the study are included in the article/supplementary material, further inquiries can be directed to the corresponding authors.
